# A 7-Year Trend of Malaria at Primary Health Facilities in Northwest Ethiopia

**DOI:** 10.1155/2020/4204987

**Published:** 2020-01-29

**Authors:** Ayenew Addisu, Yalewayker Tegegne, Yenesew Mihiret, Abebaw Setegn, Ayalew Jejaw Zeleke

**Affiliations:** Department of Medical Parasitology, School of Biomedical and Laboratory Sciences, College of Medicine and Health Sciences, University of Gondar, Gondar, Ethiopia

## Abstract

**Background:**

Malaria is a severe parasitic disease that can progress to complications of the nervous system, respiratory distress, renal problems, metabolic acidosis, and hypoglycemia which can result in death in case of delay or absence of appropriate treatment. Even though health service facilities and vector control strategy in the community are implemented as control measures, variations in temperature and rainfall that can affect the life cycle of parasite are among the factors of malaria prevalence over the years. The aim of this trend analysis was to assess the prevalence and the impact of malaria over the seasons and years.

**Methods:**

A cross-sectional study using retrospective information was conducted at two health centers Gorgora and Chuahit in Dembia district. The data was collected from lab logbooks routinely diagnosed and registered for seven years. A systematic sampling technique was used by taking patient results from lab logbooks during the first ten days of every month. Data were entered directly into the EpiData Entry software version 3.1 and analysed with the SPSS software version 20. Moreover, a chi-square test with a level of significance set at less than 5% was used.

**Results:**

From a total of 11,879 clients that participated, 56.6% were males. The overall malaria prevalence in the last seven years was 21.8%, and the dominant parasite was *P. falciparum* which accounted for 15.6% of the participants which was threefold higher than *P. vivax* in the seven-year trend. Moreover, at Gorgora health center, the prevalence which was 15% at the beginning of the study (2012) rose to 33.9% and 30.5% in 2017 and 2018, respectively. In the analysis of the seven years, October and September in which the prevalence of malaria was 32.6% and 27.2%, respectively, constituted the peak months. High malaria prevalence was observed in autumn (September to November) season, and the least was observed in spring (March to May) with the prevalence of (17.8%) (*p* ≤ 0.001). Malaria attack showed significant variability among different age groups, and the age group 15-29 and males were the most affected (*p* ≤ 0.001).

**Conclusion:**

In this study, malaria transmission remained high, which affected males more than females. Thus, appropriate season-based bed net use, health education, immediate patient treatment, and stagnant water drainage methods are needed to alleviate the problem.

## 1. Background

Malaria is a life-threatening protozoan disease caused by plasmodium parasites that transmit to people through the bites of infected female *Anopheles* mosquitoes. Five Plasmodium parasitic species cause malaria in humans; of these, two species, *Plasmodium falciparum* and *Plasmodium vivax*, are predominant [1–3].

Malaria is a severe disease, which even progresses to complications and death if there is a delay or absence of appropriate treatment. Some of the clinical complications and manifestations observed in malaria infection are nervous system involvement, respiratory distress, renal problems, metabolic acidosis, and hypoglycemia [4].

Malaria infection affects nearly half of the world's population. As per the 2018 World *malaria report*, there were 219 million malaria cases, of which the 10 highest victims were African countries. It affects the lives of more than 435,000 people, the majority of whom are in Africa each year. Pregnant women and children under five years are among the most vulnerable groups [5]. To reduce malaria mortality in 15 high-burden sub-Saharan countries by 50%, it requires an artemisinin-based combination therapy to create malaria-free zones in Africa by 2040-2050 [6].

In Ethiopia, the overall trends of malaria cases and deaths among under-five children decreased by 59.3% and 72.1%, respectively. On the other hand, malaria incidence rate decreased from an average of 43.1 to 29 cases and 2.1 to 1.1 deaths per 1000 people annually between 2001 and 2016 [7]. According to the 2016 Ethiopian malaria intervention report, there were 64% and 92.5% coverage of indoor residual spray (IRS) and insecticide-treated net (ITN), respectively [7].

Malaria transmission control strategies are very complex issues, influenced by various factors that may relate to the host, the parasite, the environment, the vector, and the health system capacity to fully implement the available rapid diagnostic tests and professional skill in microscopic species detection [5]. Besides, the most effective prevention and control strategies are active case management, operational research and surveillance, and monitoring and evaluation systems to provide appropriate information [5].

Therefore, the prevalence of malaria is expected to shift over the years because of the increment in the number of health service facilities, high vector control strategy coverage by using indoor residual spray and insecticide-treated nets in the community, and the awareness of the rural population about health seeking behavior [8]. Besides this, to accelerate malaria control strategy, considering seasonal malaria prevalence variability over the years is crucial.

Information from specific health facility laboratory registers over the long periods of time is valuable for a better malaria control strategy. Therefore, this long-term malaria parasite trend over the years and the variability within a year in laboratories of two primary health facilities have provided valuable information for further action.

## 2. Methods

### 2.1. Study Area, Population, and Period

Dembia district is located in the Amhara region of Ethiopia 35 km from the Gondar town. It was bordered by Gondar town and Takusa, Chilga, and Belesa districts. Dembia has a total population of 307,967 in an area of 148,968 sq. km. Its altitude ranges from 1, 850 to 2, 000 m above sea level with an average annual rainfall of 700 to 1, 160 mm. It has 10 health centers and 40 health posts. Among the ten health centers, Gorgora and Chuahit were chosen for this study. Each health center was 16 km apart, and Gorgora was bordered by Lake Tana. The study participants were all clients who provided blood samples for laboratory blood film microscopic examinations and registered on the lab logbooks of the two health centers [9, 10].

### 2.2. Study Design, Sampling Technique, and Data Collection

A cross-sectional study using retrospective information was carried out in the two health centers of Dembia district. The data was collected from seven years of registered lab logbook that stained and diagnosed with Giemsa's staining technique. Since taking the whole examined blood films over seven years in the two health centers is cumbersome, we systematically took patient results of the first ten days of every month.

### 2.3. Analysis and Statistics

Data from the laboratory registered logbook was entered directly into the EpiData Entry software version 3.1 and analysed with the SPSS software version 20. Excel was also used to summarize some figures. Descriptive statistics were used to summarize the data and to display malaria parasite trends over the years and seasonal variations. We compared parasite proportions by gender, age groups, seasons of the year, and trends over the years. Moreover, a chi-square test with a level of significance set at less than 5% was considered.

## 3. Results

A total of 11,879 clients were requested for blood film and registered on lab logbooks of the two health centers. Of whom, 6, 724 (56.6) were males and 7, 859 (66.2%) were enrolled from Chuahit health center. The total malaria prevalence over the seven years was 21.8%, of which *P. falciparum* that accounted for 15.6% was the dominant parasitic species, followed by 733 (5.3%) of *P. vivax*. The rest 101 (0.9%) were mixed or both *P. falciparum and P. vivax* infections.

### 3.1. Malaria Trend in Different Years and Seasons

**Figure 1:**
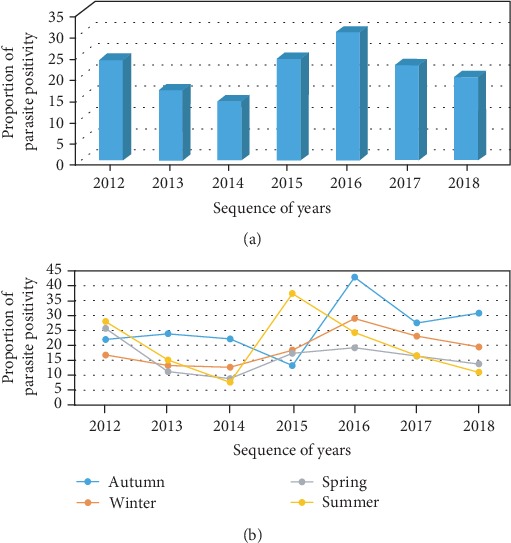
Seasonal variations and trend prevalence of malaria infection over the study period in Dembia district health centers from 2012 to 2018. (a) Annual trend prevalence. (b) Positivity rate across seasons (*n* = 11,879).

Over the last seven years, the prevalence of malaria was highly variable across years ranging from 14% to 30.2% (*p* ≤ 0.001). The highest prevalence was observed in 2016 (30.2%) followed by 2015 (24%). Of the twelve months of the seven years, October had the highest prevalence (32.6%) followed by September (27.2%). On the other hand, the least prevalence was observed in February (15.1%). As far as malaria prevalence across the different seasons was concerned, the highest prevalence was observed during autumn (September to November) (27.9%), followed by 23.3 and 18.4% in summer (June to August) and winter (December to February), respectively. Spring (March to May) was the season with the least prevalence (17.8%) (*p* ≤ 0.001) (Figure 1).

### 3.2. Malaria Distribution in relation to Age and Sex

**Figure 2:**
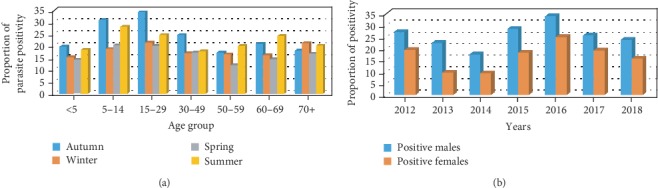
Malaria infection in different seasons with age groups and sex in Dembia district health centers from 2012 to 2018. (a) Malaria prevalence in different seasons with age groups. (b) Sex and malaria prevalence distribution (*n* = 11,879).

The prevalence of malaria varied among different age groups ranging from 16.3% to 25.5% (*p* ≤ 0.001). The 15-29-year age group was the most affected followed by the 5-14-year group with the prevalence of 25.5% and 24.6%, respectively. The least malaria prevalence was observed among 50-59 years of the age group (16.3%). In this study, males were more affected than females over the seven years, which ranged from 17.4% to 33.7% as shown in Figures 2(a) and 2(b).

### 3.3. Plasmodium Species Distribution among People and Health Centers

**Figure 3:**
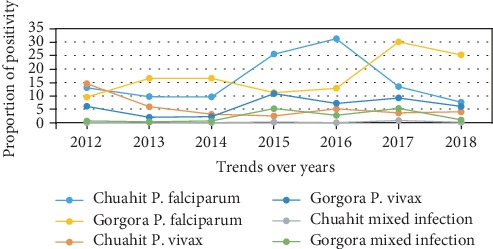
Plasmodium species distribution on the population and health centers in the last seven years in Dembia district health centers from 2012 to 2018.

*P. falciparum* was threefold more dominant than *P. vivax* in the seven-year trend (15.6% and 5.3%, respectively). Moreover, at Gorgora health center, the prevalence which was 15% at the beginning of the study (2012) rose to 33.9% and 30.5% in 2017 and 2018, respectively. On the other hand, at Chuahit health center, the prevalence decreased from 27.4% in 2012 to 11.6% at the end of 2018, although after high prevalence was seen in 2016 (Figure 3).

In the study district, long-lasting insecticidal nets (LLIN) were distributed in proportion to family members every three years. Carbamate group chemicals bendiocarb and propoxur wettable powders were used to control mosquito vectors over the last seven years. Either one of the two or combined composition of bendiocarb and propoxur chemicals was for indoor residual spray to control mosquito landing.  Table 1 shows the houses sprayed for seven years and population expected to have been protected.

## 4. Discussion

The purpose of this trend analysis was to investigate the spread and impact of malaria across the nations of Ethiopia and seasons from 2012 to 2018 in order to highlight priorities for the Ethiopian Vector-borne Disease Control Program intended to free the nation from the disease.

According to this study, the trend of the prevalence of malaria among the 11,879 clients who were registered in the lab logbooks of the two health centers and requested for blood films over the seven years was 21.8%. This result was markedly lower than 36.1%, 48%, and 33.8% reported from the nearby localities of Addi Arkay health center, northwest Ethiopia; Woreta town, northwest Ethiopia; and Abeshge, south-central Ethiopia, respectively [8, 11, 12]. On the other hand, it was higher than the five-year trend study conducted in Ataye, North Shoa, Ethiopia; reported a total prevalence of 8.4%, the seven-year retrospective malaria report from Metema hospital, northwest Ethiopia; detected a prevalence of 17%, the fifteen-year study in Ethiopia; and noted a prevalence of 12.5% [13, 14]. The differences might be due to time variations of the studies, difference in insecticide application in the areas, variations in geographical locations, differences in population awareness about malaria bed net application, its transmission, and health seeking behavior.

During the study, the highest prevalence 30.2% was observed in 2016, followed by 2015 (24%). These results were similar to the results of a study on malaria epidemiology and interventions in Ethiopia which reported a decrease in malaria incidence and death rate in Ethiopia from 2001 to 2016. But malaria case number and incidence reported in 2016 exceeded the WHO standard for “pre-elimination strategy” [7]. This might be due to the use of propoxur instead of bendiocarb as indoor residual spray insecticide or relatively decreased number of subcities covered by chemical spray (Table 1).

In the current study, the prevalence of malaria was high in October and the least in February over the seven years of twelve months. Seasonal fluctuations and high prevalence of malaria were observed in autumn (September to December) and the least prevalence in spring (March to May). This was in line with the findings of studies conducted in Ataye, North Shoa, Ethiopia (by Ethiopian Federal Ministry of Health); Addi Arkay health center (Ethiopia); Abeshge (south-central Ethiopia); and Metema hospital, northwest Ethiopia [8, 11, 14]. Such high malaria prevalence in autumn (September to December) might be related to the formation of stagnant water after the heavy rain season, favorable temperature, and high vegetation density for mosquito breeding. On the other hand, the least prevalence was observed in spring (March to May); this might be due to drought.

The study also showed that the prevalence of malaria was high among males and 15–29 years of age groups over the seven years trend. This was similar to the findings of studies conducted in Addi Arkay health center; Ataye district, North Shoa; and Abeshge, south-central Ethiopia [8, 11, 14]. This might be due to the fact that productive age groups often engaged in farm activities in the fields. Males usually sleep outdoors to look after farms. In most Ethiopian communities' culture, females are restricted to home activities, like taking care of children and usually spend their time indoors.

Moreover, *P. falciparum* was more dominant parasite than *P. vivax* which it exceeded by over threefold. The finding was comparable with the results of studies in Addi Arkay, northwest Ethiopia; Ataye, North Shoa, Ethiopia (by Ethiopian Federal Ministry of Health); Abeshge, south-central Ethiopia; Woreta town, northwest Ethiopia; and Metema hospital, northwest Ethiopia, which reported that *P. falciparum* was the dominant parasite over the study periods of each study [11–15]. The reason may be that the *P. falciparum* parasite can multiply rapidly by involving more than one parasite in a single red blood cell, colonizing all ages of the red blood cells without any selection, parasite-infected red blood cells (RBCs) can accumulate in various organs, and the availability of *P. falciparum* infected cases in communities [16, 17].

Malaria was more widespread in Gorgora health center compared to Chuahit over the seven years; the prevalence at the beginning of the study was 15% and the peak was 33.9% in 2017. At Chuahit health center, the prevalence declined from 27.4% in 2012 to 11.6% in 2018. This might be due to geographical location as Gorgora is located nearer Lake Tana; the vegetation in the shore may be appropriate for mosquito breeding.

In this study area, long-lasting insecticidal nets (LLIN) and IRS were used, though the coverage of IRS was low. This result was similar to the result of a study conducted by the Malaria Epidemiology and Interventions in Ethiopia, and it was reported that IRS coverage was low nationwide with an average coverage of 23% between 2014 and 2016 [7].

Data collection in the two health centers on the whole examined blood films over seven years was cumbersome; we systematically took patient results of the first ten days of every month. Moreover, the collected data relayed on the laboratory logbook which lacks participants' body temperature and clinical presentations were the main limitations of the study.

## 5. Conclusion

In this study, malaria transmission remains high, especially in autumn and summer seasons. The most affected were the productive age groups with a high infection rate in males. The dominant parasite was *P. falciparum*; hence, appropriate vector control methods that target outdoor and indoor transmissions are needed, especially for males during high malaria transmission seasons to avoid factors that affect success of malaria elimination.

## Figures and Tables

**Table 1:** Insecticide distribution, number of sprayed houses, and number of protected population in Dembia district from 2012 to 2018.

Study years	No. of sprayed houses	Population protected against mosquitoes	Total population	No. of subcities covered by chemical spray	Types of chemicals used
2012	45,351	250,530	295,802	47	Propoxur and bendiocarb
2013	76,033	259,987	301,073	42	Bendiocarb
2014	46,494	179,566	309,458	29	Bendiocarb
2015	22,623	84,706	316,913	15	Bendiocarb
2016	52,397	181,704	321,877	29	Propoxur
2017	13,911	47,077	328,757	7	Propoxur
2018	87,665	163,899	335,637	21	Bendiocarb

## Data Availability

All data generated or analysed during this study were included in this published article.

## Abbreviations

IRS: Indoor residual spray
ITN: Insecticide-treated net.
